# Unmethyl-esterified homogalacturonan and extensins seal *Arabidopsis* graft union

**DOI:** 10.1186/s12870-019-1748-4

**Published:** 2019-04-18

**Authors:** Katarzyna Sala, Jagna Karcz, Aleksandra Rypień, Ewa U. Kurczyńska

**Affiliations:** 1 Department of Cell Biology, Faculty of Biology and Environmental Protection, University of Silesia in Katowice, Jagiellońska 28 St, 40-032 Katowice, Poland; 2 Laboratory of Microscopy Techniques, Faculty of Biology and Environmental Protection, University of Silesia in Katowice, Jagiellońska 28 St, 40-032 Katowice, Poland

**Keywords:** *Arabidopsis*, Seedling grafting, Histology, Cell wall epitopes, Homogalacturonan, Extensins, SEM

## Abstract

**Background:**

Grafting is a technique widely used in horticulture. The processes involved in grafting are diverse, and the technique is commonly employed in studies focusing on the mechanisms that regulate cell differentiation or response of plants to abiotic stress. Information on the changes in the composition of the cell wall that occur during the grafting process is scarce. Therefore, this study was carried out for analyzing the composition of the cell wall using *Arabidopsis* hypocotyls as an example. During the study, the formation of a layer that covers the surface of the graft union was observed. So, this study also aimed to describe the histological and cellular changes that accompany autografting of *Arabidopsis* hypocotyls and to perform preliminary chemical and structural analyses of extracellular material that seals the graft union.

**Results:**

During grafting, polyphenolic and lipid compounds were detected, along with extracellular deposition of carbohydrate/protein material. The spatiotemporal changes observed in the structure of the extracellular material included the formation of a fibrillar network, polymerization of the fibrillar network into a membranous layer, and the presence of bead-like structures on the surface of cells in established graft union. These bead-like structures appeared either “closed” or “open”. Only three cell wall epitopes, namely: LM19 (un/low-methyl-esterified homogalacturonan), JIM11, and JIM20 (extensins), were detected abundantly on the cut surfaces that made the adhesion plane, as well as in the structure that covered the graft union and in the bead-like structures, during the subsequent stages of regeneration.

**Conclusions:**

To the best of our knowledge, this is the first report on the composition and structure of the extracellular material that gets deposited on the surface of graft union during *Arabidopsis* grafting. The results showed that unmethyl-esterified homogalacturonan and extensins are together involved in the adhesion of scion and stock, as well as taking part in sealing the graft union. The extracellular material is of importance not only due to the potential pectin–extensin interaction but also due to its origin. The findings presented here implicate a need for studies with biochemical approach for a detailed analysis of the composition and structure of the extracellular material.

**Electronic supplementary material:**

The online version of this article (10.1186/s12870-019-1748-4) contains supplementary material, which is available to authorized users.

## Background

Grafting is a technique in which either organs from different plants (heterografting) or from the same plant (autografting) are joined so as to continue their growth together. The upper part of the combined plant is called the scion, while the lower part is called the rootstock. In addition, the isolated organ fragments [1], or even callus tissues [2, 3], can be successfully used for grafting. Although grafting has been performed for centuries, the mechanisms that regulate this process are still unclear (for a review see [4]). In the last ten years, there has been growing interest in grafting especially with respect to hormonal and genetic analyses of vascular regeneration, reactions to wounding, and cell differentiation, and recent studies have provided new information using mutants and transgenic lines of different species, as well as with the help of new research techniques [5–8].

Grafting leads to the development of a stable union zone in which a structural and functional connection between the vascular system and other tissues of the scion and rootstock is established [9]. A stable graft union is achieved with the formation and differentiation/re-differentiation of the callus cells of scion and stock. Various differentiation processes that occur during regeneration result in differences in the arrangement of the cells/tissues and in cell phenotypes in the union zone compared to the “mother” parts of the grafted scion and stock [10].

Before the above-described cell events occur, an adhesion between the scion and the stock develops and stabilizes [5, 11]. During the first stage of grafting, the callus emerges as a result of wounding [12], and subsequently non-sister cells adhere de novo at the graft interface. On the surface of the callus cells, numerous bead-like structures, consisting of carbohydrates (mainly pectins) and proteins, appear [12–14]. Thus, during the “recognition” of the callus cells from the scion and rootstock, different cell wall components may be observed [15].

Pectins comprise a heterogeneous group of polysaccharides composed mainly of galacturonic acid residues [16]. Within the pectin “family”, the homogalacturonan (HG) and rhamnogalacturonan I (RG I) domains can be clearly distinguished. HG domains are believed to be the most widespread and constitute up to 65% of all cell wall pectins [17]. HG is synthesized and incorporated in the cell wall in a methyl-esterified form [18]. Removal of methyl esters from the cell wall matrix results in varied degree and pattern of methyl esterification which, in turn, is reflected in the different properties of HG (e.g. rheological properties) [19, 20]. In contrast to HG, the amount and distribution of RG I domains are thought to be variable and undergo dynamic changes during development of cells or tissues. A previous study has shown that changes in the composition of side chains, which are composed of neutral sugars like galactan or arabinan, are correlated with the status of cell differentiation and determine the mechanical properties of the cell wall [21]. Alike pectins, hemicelluloses are a heterogeneous group of polysaccharides, and their structure, occurrence, and function vary depending on the type of plant species, tissues, and cells [22]. Apart from their main structural role, where hemicelluloses together with cellulose microfibrils form a scaffold responsible for the mechanical properties of the cell wall, these polysaccharides may also serve as a storage material for plant cells [23, 24]. It is interesting that oligosaccharides that originate from the enzymatic or mechanical defragmentation of the polymers in the cell wall, such as xyloglucan [25] or HG, are biologically active fragments and act as endogenous growth regulators that trigger physiological reactions [26, 27].

Arabinogalactan proteins (AGPs), a class of highly glycosylated proteins [28–31], have been detected in many cellular compartments, including cell wall matrix, cell membrane, tonoplast, and vacuole, and also in various cellular secretions [32–35]. The localization of these proteins and their transient presence in various cellular compartments suggest that they are involved in cell signaling rather than being a structural component [28, 34, 36–38] and may act as factors triggering plant cell differentiation [30]. Another class of cell wall proteins, called extensins, have a protein core that is mainly rich in hydroxyproline and serine; hence, this class of proteins are also known as hydroxyproline-rich glycoproteins (HRGPs) [28]. Unlike AGPs, which can be easily extracted from the cell wall and which are considered to be “mobile”, extensins are extremely resistant to extraction, and even after secretion into the cell wall, they immediately become immobilized via covalent bonding with other extensin molecules [39, 40] or other polymers in cell wall, presumably pectins [41], thereby forming networks that influence the extensibility of cell wall. Increase in the content of extensins in the cell wall results in termination of cell growth [28, 42, 43]. Moreover, the amount of extensins significantly increases in plants following mechanical injury or pathogen attacks [44–46].

Understanding the roles of the cell wall components in plant cell differentiation processes is crucial for enhancing the knowledge base and for their commercial applications. While studying the changes in cell wall composition that occur during grafting of the *Arabidopsis* hypocotyl, we observed the formation of a layer covering the surface of the graft union. As this phenomenon has not been described so far, we focused on the exterior area of a graft union instead of the adhesion zone, which has been the subject of numerous studies. The aims of this study were 1) to describe the histological and cellular changes that occur during the process of regeneration in autografted *Arabidopsis* hypocotyls and 2) to perform preliminary chemical and structural analyses of the material that extracellularly deposits and finally seals the graft union.

## Results

### Formation of the graft union – morphological features

**Fig. 1:**
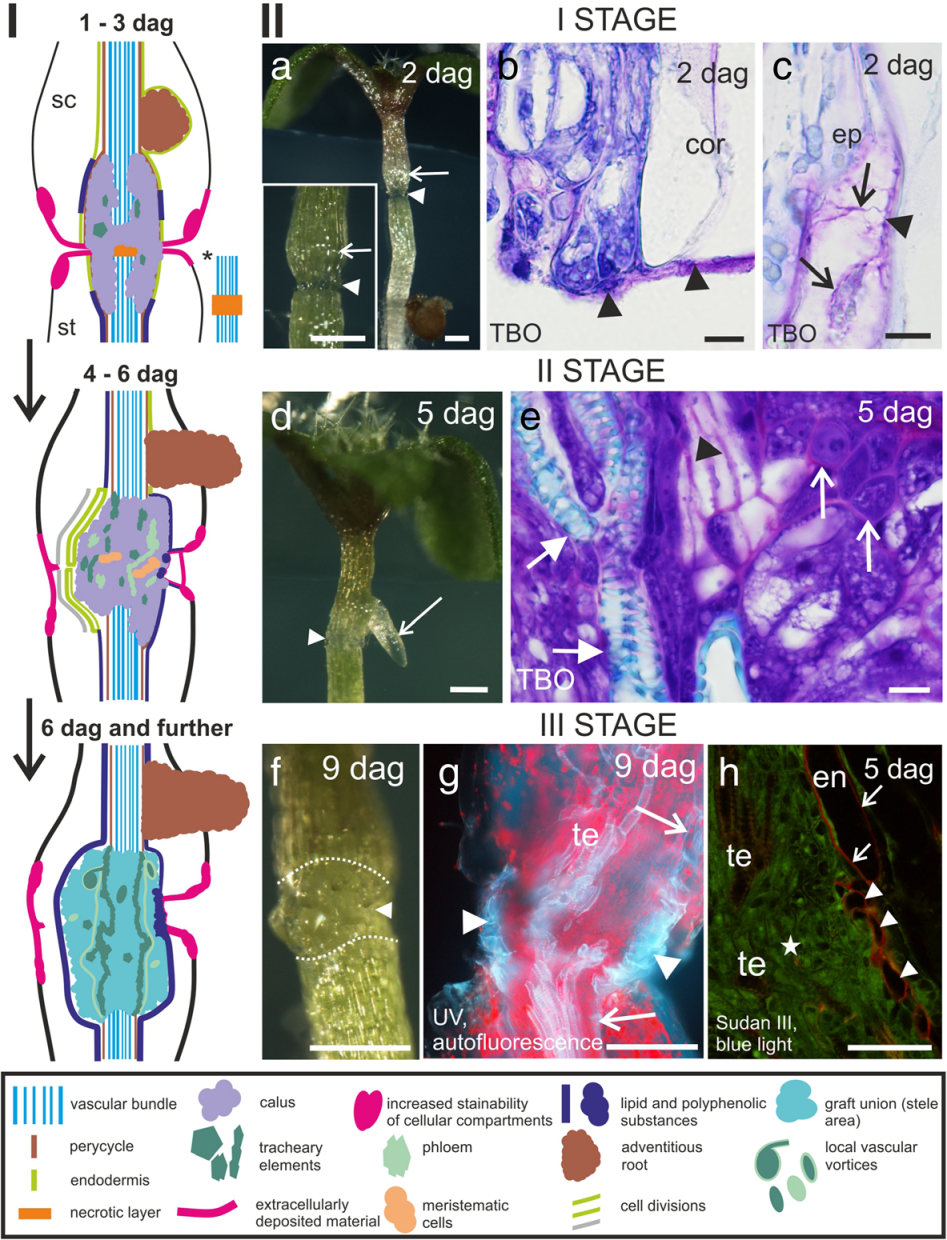
Hypocotyl grafting: (I) schematic representation of the following stages and (II) important morphological features. **a** and *inset* – graft union with callus cells (arrowheads), local increase in the circumference of the scion (arrows). **b** – scion, cut surface covered with extracellular material (arrowheads). **c** – epidermal cells, increased staining of the cytoplasmic compartments (arrows and arrowhead). **d** – stable graft union (arrowhead), developing adventitious root (arrow). **e** – tracheary elements (full arrows), sieve elements (arrowhead), and meristematic cells (arrows). **f** – graft union (arrowhead), dotted line – site of the cut. **g** – bluish autofluorescence of polyphenolic compounds present in the walls of the endodermal (arrows) and peripheral cells of the graft union (arrowheads); pink – autofluorescence of chlorophyll. **h** – lipid substances stained red and detected in the walls of the endodermal (arrows) and peripheral cells of the graft union (arrowheads), asterisk – graft union area. **Cor** cortex, **dag** days after grafting, **en** endodermis, **ep** epidermis, **sc** scion, **st** (root)stock, **TBO** toluidine blue O, **te** tracheary elements. Scale bars: a, a *inset*, d, and f = 200 μm; g = 100 μm; h = 50 μm; **b**, c, and e = 10 μm

Three time frames were chosen to evaluate the process of regeneration of the *Arabidopsis* hypocotyls during grafting based on the occurrence of dominant cellular events (Fig. 1, section I). The first time frame, that is, 0–3 days after grafting (dag), was characterized by a fragile graft union zone (scion and stock came apart during the preparation or fixation procedure). During this stage, an increase in the scion circumference was observed (Fig. 1a), callus cells were found to emerge, and the cut surface of the scion and stock was covered by an extracellularly deposited material (Fig. 1b). Adventitious roots were also found to develop (not shown). An increased stainability was observed in the intercellular spaces and in the walls of cortical cells (not shown), as well as in the cytoplasmic compartments of some of the cortical and epidermal cells that were located near the site of the cut (Fig. 1c). During the second time frame (i.e., 4–6 dag), the graft union was found to be more stable and the adventitious roots were also developed further (Fig. 1d). The surface of the scion and stock was still covered with the extracellular material. Differentiated tracheary and sieve elements and groups of meristematic cells were observed within the graft union zone (Fig. 1e). In the third time frame (i.e., 6 dag and further), the graft union was found to be filled with cells (Fig. 1f) and the vascular system was reconnected (Fig. 1g). In addition, polyphenolic and lipid substances were detected in the walls of the cells that were adjacent to the regenerated stele (Fig. 1g and h).

### Established graft union – appearance of extracellular material and bead-like structures

**Fig. 2:**
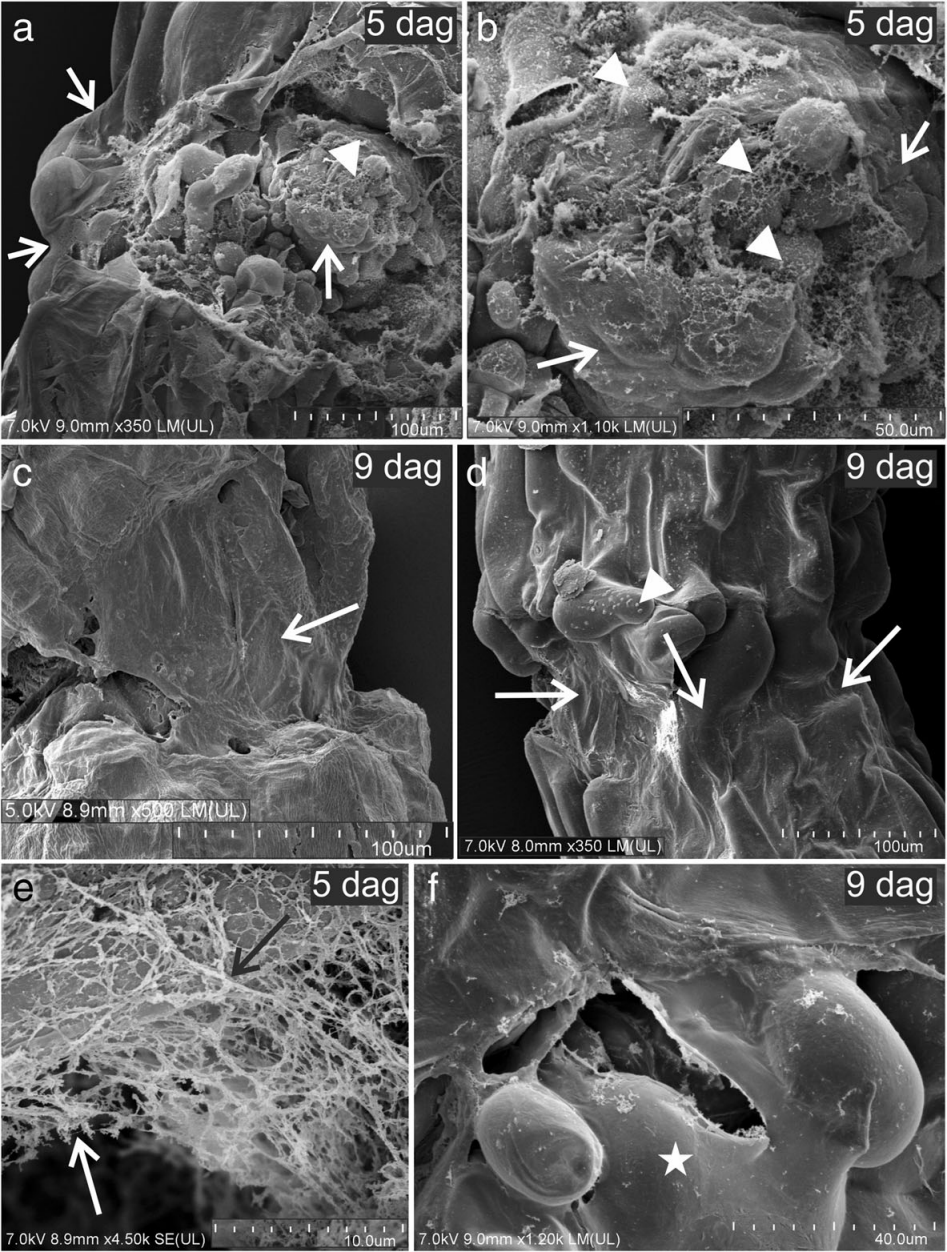
SEM images of graft union. **a** and **b** – some of the callus cells from the graft union covered with a membranous structure (arrows) and some covered with a fibrillar material (arrowheads). **c** and **d** – the graft union enveloped with a membranous structure (arrows), arrowhead – cell not covered with a membranous structure, visible bead-like structures on the surface. **e** – net-like structure formed from the fibrillar material (arrows). **f** – membranous structure (asterisk) that covers the graft union cells. **Dag** days after grafting

Scanning electron microscope (SEM) analysis showed that extracellular deposition of material accompanied the formation of graft union during all the stages of regeneration. During the second time frame (4–5 dag), material that was deposited on the surface of the callus cells appeared in two structural forms: fibrillar and membranous (Fig. 2a, b, and e). The terms “fibrillar” and “membranous” are only used to describe the physical shape of the deposited material and do not refer to the chemical nature of its components (although the further analyses pointed to them as carbohydrates/proteins). Both fibrillar and membranous forms were observed covering the groups of callus cells (Fig. 2a and b). In the older grafts, that is, during the third time frame (8–9 dag), the graft union zone was found to be sealed by a membranous structure (Fig. 2c and d). The border between the stock–scion epidermis and the graft union cells was barely visible (Fig. 2c and d). In addition, some bead-like structures were observed on the surface of callus cells that were not covered by a membranous layer (Fig. 2d). The fibrillar material that was observed during the second time frame had a net-like structure (Fig. 2e); however, it was rarely found in the older grafts (Fig. 2f). Thus, it can be suggested that the formation of fibrillar material precedes the formation of membranous structure, which might arise through polymerization or other processes of conversion of the fibrillar material.

**Fig. 3:**
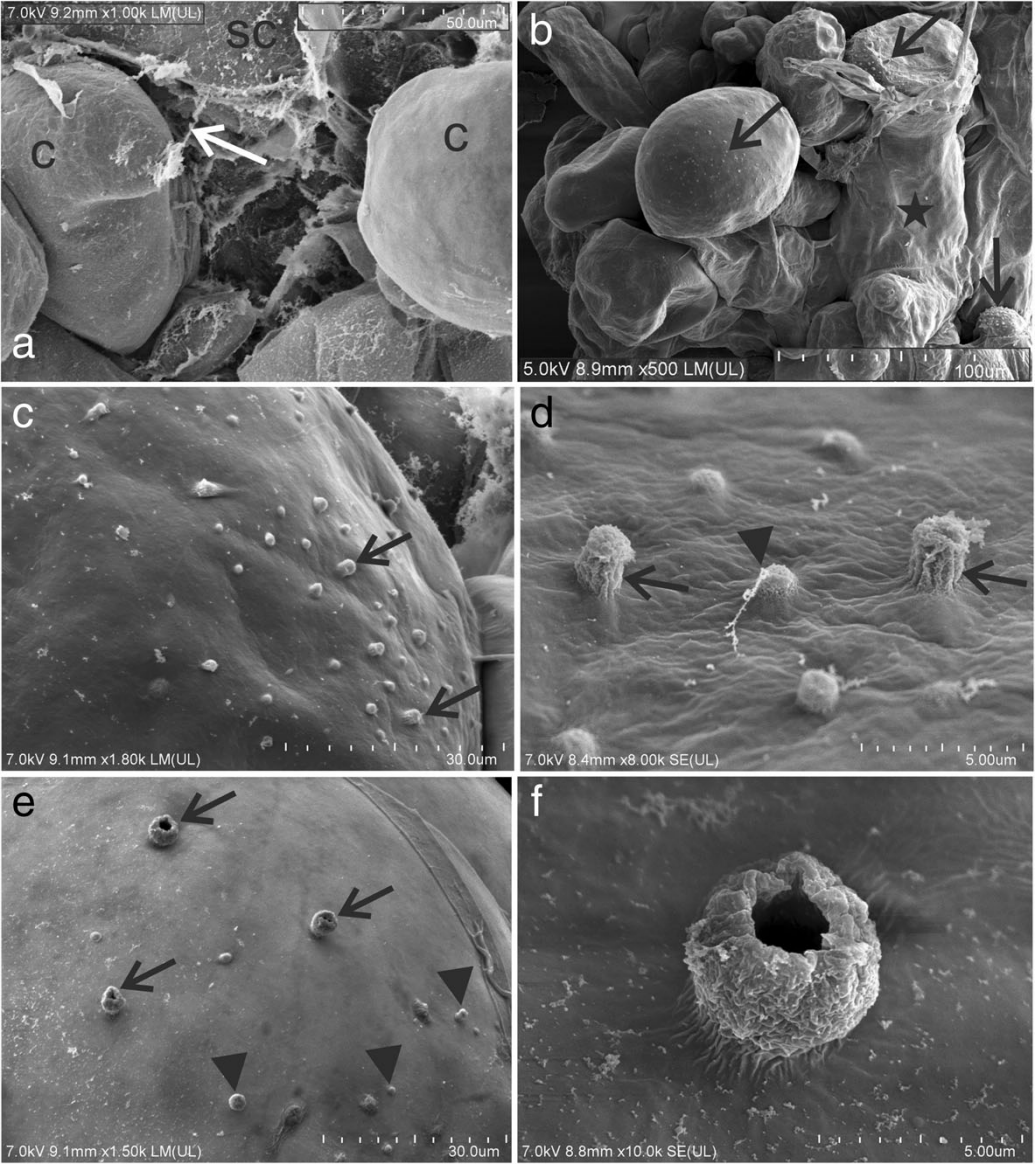
SEM images of cells from graft union. **a** – callus cells (**c**) without bead-like structures, fibrillar material associated with some of them (arrow). **b** – graft union, bead-like structures on the surface of the cells that are not covered with a membranous structure (asterisk). **c** and **d** – bead-like structures on the cell surface (arrows), arrowhead – fibrillar material associated with some of the bead-like structures. **e** – two types of bead-like structures found: “closed” (arrowheads) and larger, “open” (arrows) ones. **f** – magnification of an “open” bead-like structure". **sc** scion

At the beginning of the regeneration process (i.e., during the first and second time frames), the callus cells in the graft union zone had a smooth surface with no visible bead-like structures; however, strands of fibrillar material were associated with some of these cells (Fig. 3a). In the more advanced stage, numerous bead-like structures were observed on the surface of the graft union cells that were not enclosed by a membranous layer (Fig. 3b and c). These structures were diverse in their size and form, and appeared either “closed” (Fig. 3c and d) or “open” (Fig. 3e and f). During this stage, the strands of fibrillar material were mostly found associated with “closed” bead-like structures (Fig. 3d). Because the “open” bead-like structures were larger than the “closed” ones, it can be speculated that the “open” form is the subsequent stage of the “closed” form.

### Detection of unmethyl-esterified HG and extensins at scion–stock interface

**Fig. 4:**
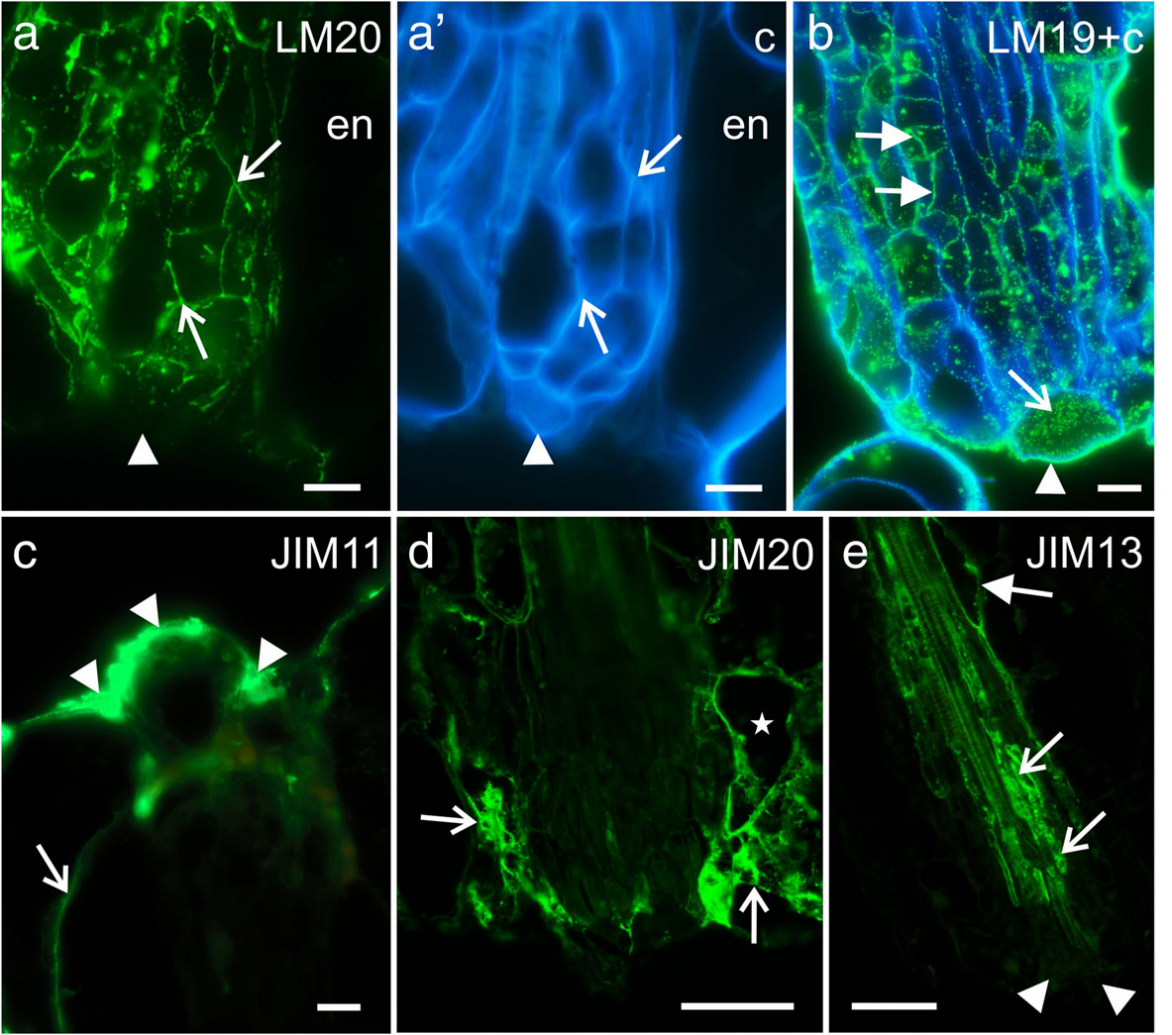
Immunohistochemistry of the grafted hypocotyl sections, first time frame – homogalacturonan (LM20 and LM19 epitopes), extensins (JIM11 and JIM20 epitopes), and AGPs (JIM13 epitope). **a** – scion, epitope present in the walls of the dividing cells (arrows), no epitope detected on the surface of the callus cell protruding outside the scion (arrowhead). **a′** a, Calcofluor White. **b** – scion, epitope present in the walls of the dividing cells (full arrows), in the cell compartments (arrow), and on the surface of the callus cells protruding outside the scion (arrowhead). **c** – stock, epitope detected in the walls (arrow) and on the surface of the cells (arrowheads). **d** – scion, epitope present in the cellular compartments of some cortical and endodermal cells, near the cut (arrows). **e** – scion, epitope present in the endodermal cells (full arrow) and cells next to the vessels (arrows), no epitope detected in the callus cells protruding outside the scion (arrowhead). **c** Calcofluor White **en** endodermis. Scale bars: a–c = 10 μm; d and e = 50 μm

In order to characterize the components of extracellular material observed during *Arabidopsis* grafting, monoclonal antibodies against cell wall components were utilized. Immunohistochemical analysis showed the abundant presence of two HG epitopes – LM19 and LM20 (un/low- and high-methyl-esterified HG, respectively) – in the walls of the dividing cells near the site of the cut; they were also seen within the maternal tissues of the scion and stock (Fig. 4a and b). However, no LM20 epitope was observed in the walls or on the surface of the callus cells that were seen protruding from the scion or stock (Fig. 4a and a'). Conversely, LM19 epitope was found abundantly in those cells, especially in the walls, in the cytoplasmic compartments, and on the surface (Fig. 4b). Similarly, two extensin epitopes (JIM11 and JIM20) were detected on the surface of the cut parts (Fig. 4c) and within the cytoplasmic compartments of some of the cortical and epidermal cells in the vicinity of the site of the cut (Fig. 4d). The extensin epitope JIM12 was not present at any of those sites (not shown). Interestingly, none of the AGP epitopes that were examined in the study were present in the callus cells or on their surface (JIM8 and LM2 are not shown; JIM13 is shown in Fig. 4e).

### Occurrence of only unmethyl-esterified HG and extensins in extracellular material and bead-like structures

**Fig. 5:**
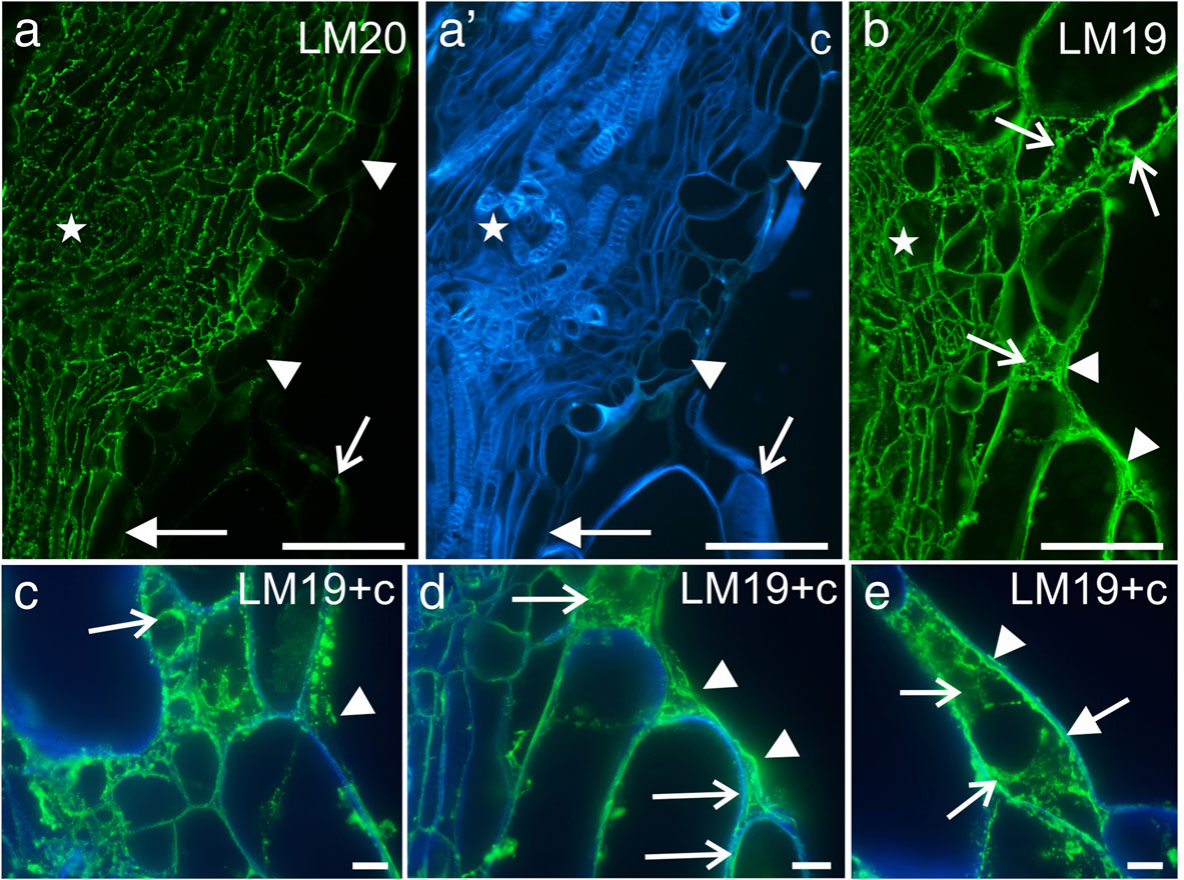
Immunohistochemistry of grafted hypocotyl sections – homogalacturonan (LM20 and LM19 epitopes). **a** – weak labeling of the epidermal and cortical cell walls (arrow), no or weak fluorescence signal in the peripheral cells of the graft union (arrowheads) and endodermal cell walls (full arrow), asterisk – graft union area. **a′** a, Calcofluor White. **b** – abundant occurrence of the epitope in the cytoplasmic compartments (arrows) and on the surface of the graft union cells located peripherally (arrowheads), asterisk – graft union area. **c** and **d** – abundant occurrence of the epitope in the cytoplasmic compartments, in the cell walls (arrows), and on the surface (arrowheads) of the graft union cells. **e** – epitope present in the cytoplasmic compartments (arrows), in the cell walls (full arrow), and on the surface (arrowhead) of the graft union cells. **c** Calcofluor White. Scale bars: a, a', and b = 50 μm; c–e = 10 μm

In the stable graft union zone, the LM20 epitope was observed in the walls of the graft union cells, except for the cells that were located peripherally (Fig. 5a and a'). Moreover, no LM20 epitope was detected on the surface of the cortical or the epidermal cells and the peripherally located graft union cells (Fig. 5a and a'). By contrast, the LM19 epitope was observed abundantly on the surface of the abovementioned cells as well as in the cytoplasmic compartments of some of these cells (Fig. 5b–e). LM7, another HG epitope (partially methyl-esterified HG), was not detected in the grafted hypocotyls at all (Additional file 1: Fig. S1A). LM8 epitope (xylogalacturonan domain in HG) was found to be present in the grafted hypocotyls; however, it was not extracellularly localized (Additional file 1: Fig. S1B and C). Although the analysis of RG I epitopes showed their presence in cell walls (LM5 epitope – galactan, LM13 epitope – processed arabinan; Additional file 2: Fig. S2A, B, E, and F) and in the cytoplasmic compartments (LM6 epitope – arabinan; Additional file 2: Fig. S2E and F) of the graft union cells, none of these epitopes was detected on the surface of graft union.

**Fig. 6:**
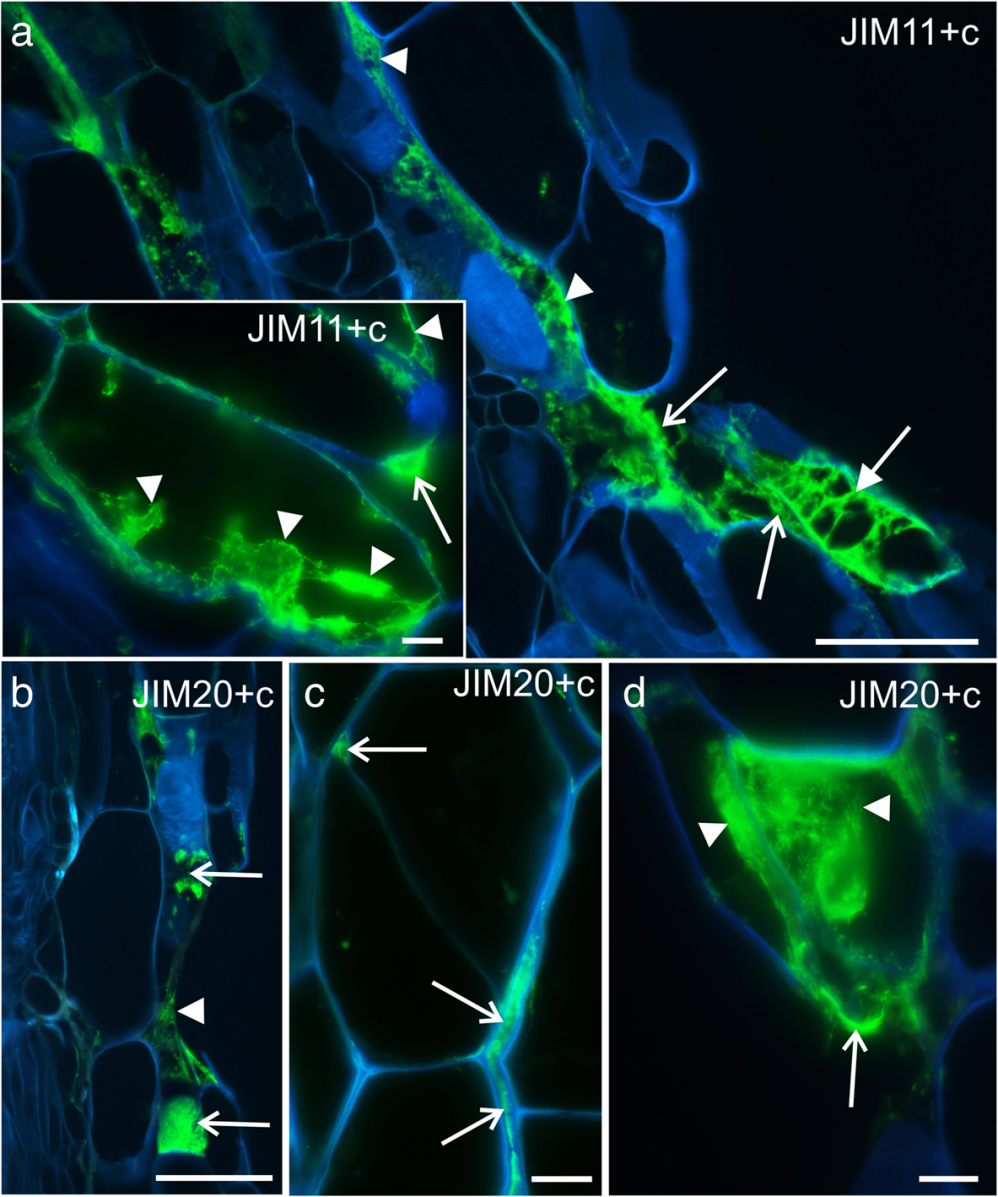
Immunohistochemistry of grafted hypocotyl sections – extensins (JIM11, JIM12, and JIM20 epitopes) and AGPs (JIM13, JIM8, and LM2 epitopes). **a** – abundant occurrence of the epitope in the intercellular spaces (arrowheads), graft union area (arrows), and cytoplasmic compartments of cells adjacent to the graft union area (arrows), *inset:* epitope present in the cytoplasmic compartments (arrowheads) and on the cell surface (arrow). **b** – abundant occurrence of the epitope in the graft union area (arrowhead) and cytoplasmic compartments of the cells adjacent to the graft union area (arrows). **c** – epitope present in the intercellular spaces between the cortical cells (arrows). **d** – scion cells adjacent to the graft union area, epitope present in the cytoplasmic compartments (arrowheads) and on the cell surface (arrow). **c** Calcofluor White, **ep** epidermis. Scale bars: a and b = 50 μm; *a inset*, c, and d = 10 μm

The occurrence of two extensin epitopes, JIM11 and JIM20, was identified to be similar to that of the LM19 epitope; these were detected on the cell surface and in the cytoplasmic compartments (Fig. 6a, b, and d). Moreover, the abovementioned epitopes were present in high amounts in the intercellular spaces that were located near the graft union zone (Fig. 6a and c). Although the third extensin epitope, JIM12, was detected abundantly in the outer periclinal walls and/or in the cuticle of the epidermis, its fluorescence signal was punctate and weak in the graft union zone compared to that of the JIM11 or JIM20 epitope (Additional file 3: Fig. S3A). The fourth epitope, LM1, was present in the walls and in the cytoplasmic compartments; however, it was found only in fewer cells compared to JIM11 or JIM20 epitope (Additional file 3: Fig. S3B and C). The epitopes of the AGPs were not found on the surface of the graft union cells located peripherally, although all the epitopes examined were detected in the cytoplasmic compartments of some of these cells (Additional file 3: Fig. S3D–F). Epitopes JIM8 (not shown) and JIM13 were also detected in the endodermal cells and in the cells associated with vascular system (Additional file 3: Fig. S3D).

The distribution of hemicellulose epitopes, LM15 (xyloglucan) and LM21 (heteromannan), during grafting process was also studied. LM15 epitope occurred abundantly in the walls of cells in the graft union area, except for endodermal or peripheral cells (Additional file 4: Fig. S4A and B), while LM21 epitope was detected in cellular compartments or, in low amounts, in the cell walls (Additional file 4: Fig. S4C and D). However, none of these epitopes was found to be extracellularly localized.

**Fig. 7:**
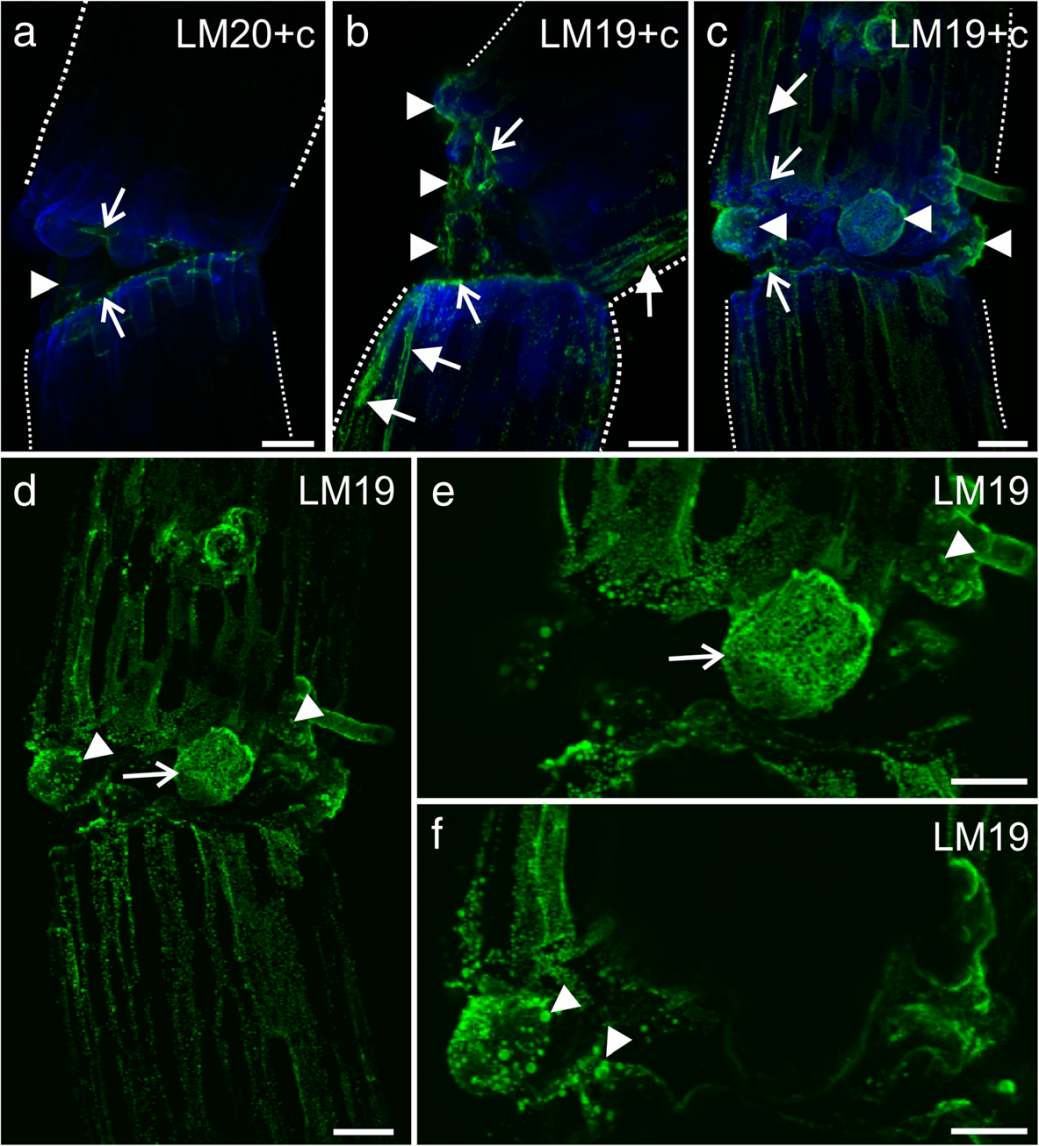
Immunohistochemistry of pectins in the grafted hypocotyls (whole mount). **a** – epitope present at the cut surface (arrows), weak fluorescence signal on the surface of the graft union cells, dotted lines – outline of the scion and stock. **b** and **c** – abundant occurrence of the epitope on the surface of the graft union cells (arrowheads), at the cut surface (arrows), and in some of the epidermal cells (full arrows), dotted lines – outline of the scion and stock. **d–f** – abundance of the epitope in the bead-like structures (arrowheads) and on the callus cell surface (arrows). **c** Calcofluor White. Scale bars: a–f = 50 μm

Results from the whole-mount immunolabeling analysis mostly correlated with those that were obtained with the stained hypocotyl sections, with the exception of the JIM12 epitope. The LM20 epitope was present at the edges of the cuts of the scion and stock, but only a weak fluorescence signal was detected in the graft union area (Fig. 7a). By contrast, the LM19 epitope was found in abundance in the graft union area (Fig. 7b–f), and a fluorescence signal was observed at the edges of the cuts of the scion and stock, as well as on the surface of the graft union cells, including the bead-like structures (Fig. 7b–f).

**Fig. 8:**
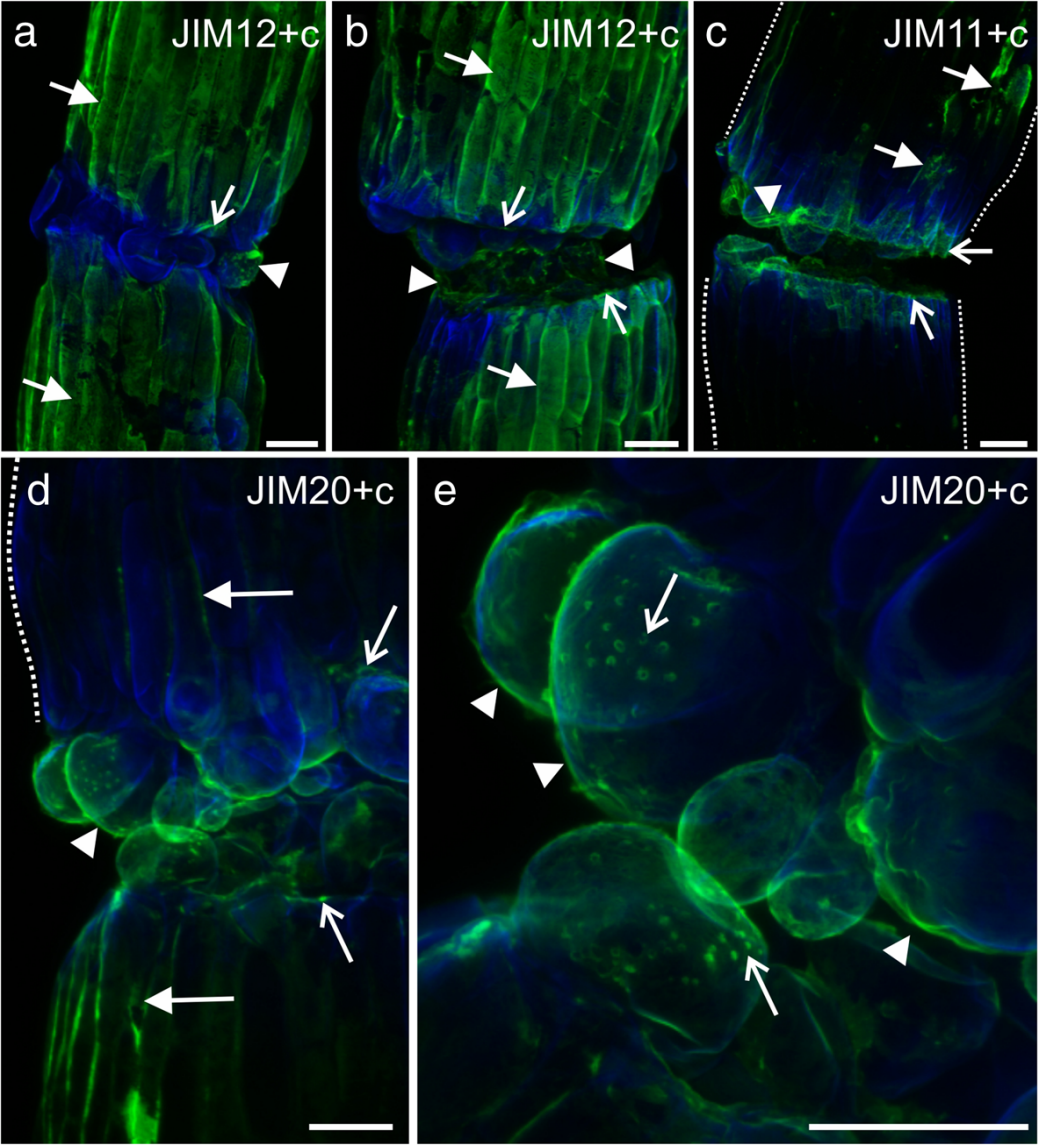
Immunohistochemistry of extensins in the grafted hypocotyls (whole mount). **a** – epitopes occurring abundantly on the surface of the graft union cells (arrowheads), at the cut surface (arrows), and in some of the epidermal cells (full arrows), dotted lines – outline of the scion and stock. **b** and **c** – epitope detected in the outer walls and cuticle of the scion and stock epidermal cells (full arrows), on the surface of the graft union cells (arrowheads), and at the cut surface (arrows). **d** – epitope occurring abundantly on the surface of the graft union cells (arrowhead), at the cut surface (arrows), and in some of the epidermal cells (full arrows), dotted lines – outline of the scion. **e** – epitope detected on the surface of the graft union cells (arrowheads) and in the bead-like structures (arrows). **c** Calcofluor White. Scale bars: a–d = 50 μm; e = 20 μm

The JIM12 epitope was primarily observed on the surface of the epidermal cells of the scion and stock (Fig. 8a and b). In some of the analyzed cells from the graft union, the JIM12 epitope was detected abundantly (Fig. 8a). However, a moderate amount of this epitope was also detected on the surface of some of the cells in the graft union (Fig. 8b). These observations differed from the results obtained from immunostaining of the sectioned material. Epitopes JIM11 and JIM20 were seen at the edges of the cuts of the two grafted parts, on the surface of the graft union cells, and especially in the bead-like structures (Fig. 8c–e). Thus, it can be declared that the JIM11 and JIM20 epitopes were observed in similar locations as the LM19 epitope.

### Results summary

**Fig. 9:**
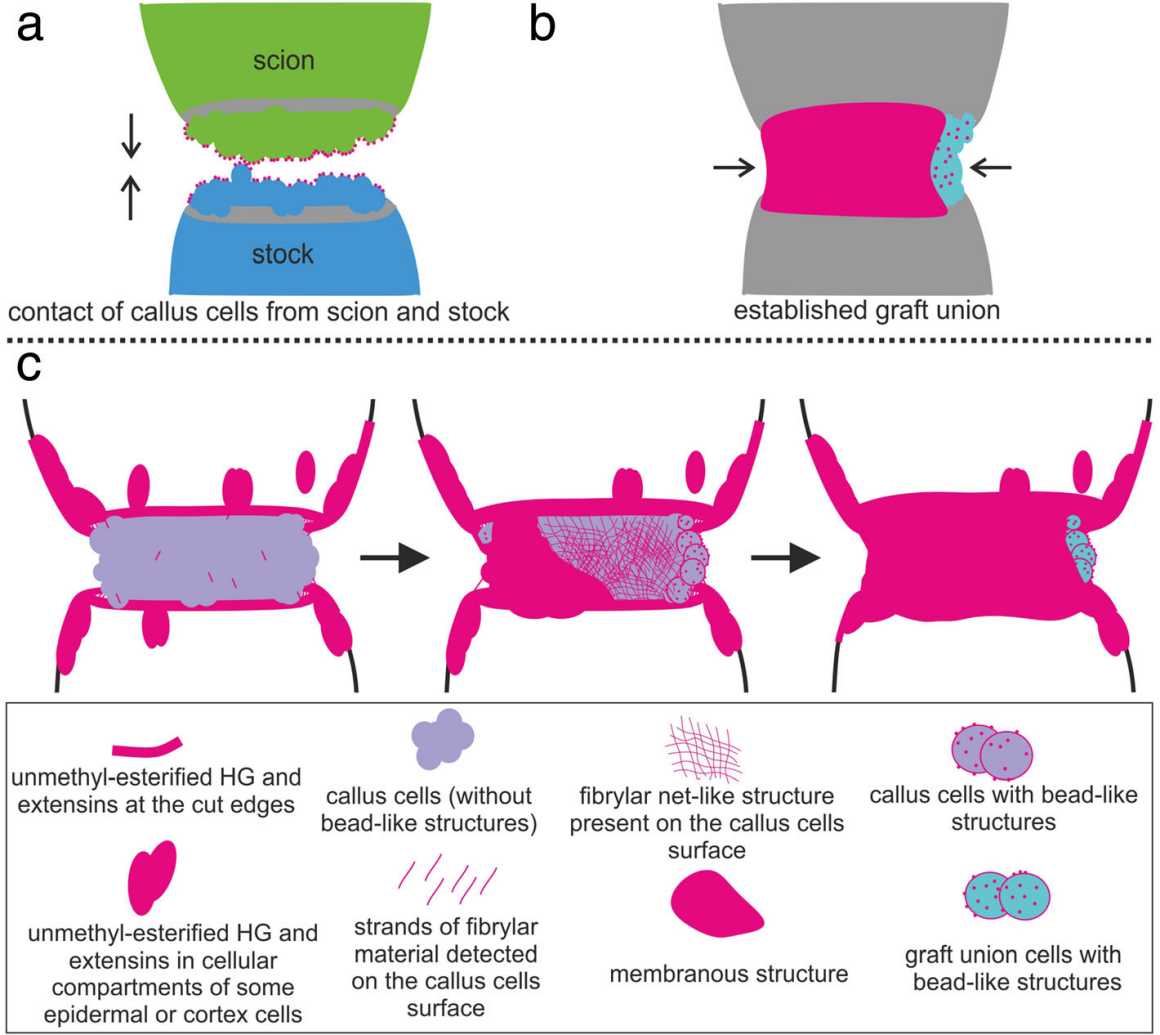
Early (**a**) and late (**b**) regeneration stages during *Arabidopsis* grafting. Schematic representation of sealing of the graft union by extracellular material

The bead-like structures, which were considered to be responsible for contact of callus cells and associated with recognition events (Fig. 9a), were found after establishment of graft union, on the surface of graft union cells, along with extracellularly deposited material that covered the graft union (Fig. 9b). The sealing of graft union with extracellular material was identified as a spatiotemporal process (Fig. 9c). In the third time frame, most of the cells were differentiated, and therefore, the term “callus” was replaced by “graft union cells” (Fig. 9c). The analysis of the 17 epitopes, belonging to different cell wall components, showed that only three were present abundantly in extracellularly deposited material and in bead-like structures (un/low-methyl-esterified HG and extensins; Table 1).

**Table 1:** Abundant (+), low (+/−), or no (−) occurrence of epitopes from different cell wall components in extracellular material and bead-like structures found in *Arabidopsis* graft union

Cell wall compounds	Occurrence in extracellular material	Occurrence in bead-like structures
Cellulose	–	–
Hemicelluloses		
Epitope recognized by LM15 antibody	–	–
Epitope recognized by LM21 antibody	–	–
Pectins		
Homogalacturonan		
Epitope recognized by LM19 antibody	**+**	**+**
Epitope recognized by LM20 antibody	+/−	–
Epitope recognized by LM7 antibody	–	–
Epitope recognized by LM8 antibody	–	–
Rhamnogalacturonan I		
Epitope recognized by LM5 antibody	–	–
Epitope recognized by LM6 antibody	–	–
Epitope recognized by LM13 antibody	–	–
Epitope recognized by LM16 antibody	–	–
AGPs		
Epitope recognized by JIM8 antibody	–	–
Epitope recognized by JIM13 antibody	–	–
Epitope recognized by LM2 antibody	–	–
Extensins		
Epitope recognized by LM1 antibody	+/−	–
Epitope recognized by JIM11 antibody	**+**	**+**
Epitope recognized by JIM12 antibody	+/−	+/−
Epitope recognized by JIM20 antibody	**+**	**+**

## Discussion

### Chemical composition of extracellular material and its implications

Among all the pectin epitopes, only two HG epitopes, LM19 (abundant) and LM20 (scarce), were detected on the surface of graft union (Table 1). Although both these antibodies can bind to the same polysaccharide domains, only LM19 can recognize unmethyl-esterified HG [47]. Because LM20 and LM7 antibodies do not bind to unesterified HG [47, 48], it can be concluded that HG detected in extracellular material is in the unesterified form. The residues of galacturonic acid present in the chains of low- or unmethyl-esterified HG can cross-link with the calcium cations, thereby creating a “pectin gel” that may cause stiffening of the cell wall [16, 49–51]. It has also been postulated that non-esterified pectins are responsible for maintaining cell adhesion. The cell-covering layer, which consists of low-methyl-esterified HG, may confine or establish a predetermined volume and limit cell division, a mechanism that was already suggested for the supraembryonic network that covers somatic embryos [52]. This layer, defined by other authors as an extracellular matrix surface network (ECMSN), has been observed on the surface of somatic embryos or embryogenic calli, or during organogenesis or callogenesis in different plant species that were cultured in vitro [53–59]. One common constituent of different ECMSNs is un/low-methyl-esterified HG, which is recognized by the LM19 or JIM5 antibodies [55, 57, 58, 60, 61]. In the present work, the occurrence of unmethyl-esterified HG may indicate a protective function that stabilizes the surface of the graft union.

The second type of chemical constituent that was found deposited outside the callus cells in the adhesion zone was extensins. Until now, the increase in extensins content in the cell wall is thought to be related to the termination of cell growth due to their involvement in the formation of the networks that stabilize the cell wall structure [42, 62]. However, recent studies have indicated that the presence of extensins may also be correlated with the increase in cell size [63] or with the initiation of cell growth [64]. It has been postulated that extensins play a role in a plant’s acquisition of resistance to pathogens (by strengthening the structure of the cell walls), and in regulation of the pectin properties and the degree of hydration of the cell wall [65–67]. In addition, extensins serve as adhesive polymers responsible for binding of cells to each other and to inert surfaces [68]. In accordance with this finding, in the current study, both unmethyl-esterified HG and extensins were detected at the scion–stock interface. Also, extensin epitopes were detected in the intercellular spaces. Some extensin epitopes (JIM11, JIM20) found in the bead-like structures were also seen in the intercellular spaces of *Asplenium*, which indicates their secretion outside the cell wall [69]. The detection of the JIM11 and JIM20 epitopes in the intercellular spaces at a distance of several rows of cells above the union zone may be interpreted that these structures resemble the secretory channel. The postulated secretion of extensins is also supported by the presence of JIM11 and JIM20 epitopes on the surface of the cells of *Arabidopsis*. It is known that in a plant organism, secretions can be collected in the extracellular spaces [70], and that some plants produce such spaces in organs that have been damaged [71, 72]. Therefore, such a location of extensins in the grafted hypocotyls confirms their participation in the plant defense reactions to damage and their protective properties against the harmful effect of the external environment.

What does finding extensins and unmethyl-esterified HG together mean? The possible interactions between HRGPs and carbohydrate polymers in the cell wall involve glycosidic linkages between arabinose or galactose from protein and sugar, covalent cross-linking to each other *via* isodityrosine residues, and formation of ionic bond between basic and acidic molecules [73]. Between both the identified components – unmethyl-esterified HG and extensins – ionic interactions can occur as they present opposite charges at physiological pH [39, 74, 75]. Basic extensins form ionic bond with acidic pectins; however, charge densities of HG may vary depending on the degree of methyl esterification, whereas extensins are only weakly charged due to their lysine content [76]. Peptide–pectin interactions can influence the properties of pectin; for example, they can reduce the swelling of pectin network in water [65]. Analysis of an in vitro biomimetic model constructed with purified molecules of extensins and pectin showed that both polymers are intimately mixed and together form a plastic network [76]. All these findings suggest that we found an interesting composite material, formed presumably by pectin–extensin network(s).

Although antibodies are a powerful tool in plant biology, the epitopes they represent are merely regions of the cell wall polymers and do not express the true heterogeneity of cell wall matrix. Thus, there is a need for further, more comprehensive approach to investigate the extracellular material, including analysis of its structure and biochemical composition, in order to find the link between its composition and its mechanical properties and function.

### Structure and origin of extracellular material

The spatiotemporal changes that were observed in the morphology of the material covering the callus and, subsequently, the graft union cells included the following: 1) formation of a fibrillar material outside the walls, 2) polymerization leading to the formation of a membranous layer, and 3) the presence of bead-like structures, which appeared either “closed” or “open”. Moreover, the structure of the extracellular material that was observed under SEM resembled an ECMSN as was mentioned earlier [52, 58].

Bead-like structures, or beads, have previously been observed on the surface of the callus cells that emerged during grafting [14, 77]. However, their occurrence was associated with the adhesion of callus of scion and stock; during contact, the bead-like structures fuse, creating the functional counterpart of middle lamellae [12]. Such structures have also been described to be related to other processes that occur during normal development [69]. In the present study, the bead-like structures were observed on the surface of the graft union cells, on different planes, and after the adhesion stage, and therefore, they may be associated with a different function. An important question is how the graft union is sealed. Hypothetically, a material containing unmethyl-esterified HG and extensins can be secreted outside of the cells by exocytosis, similar to the mucus cells. The presence of the LM19, JIM11, and JIM20 epitopes in the cytoplasmic compartments, in the cell walls, and on the surface of cell walls supports the logical sequence of the secretory pathway, thus confirming the exocytosis. However, whether the bead-like structures are an expression of the exocytosis is still unclear. Also, it is not known whether the “open” form is the later stage of the “closed” one. Furthermore, whether the occurrence of the fibrillar material on the surface of the callus cells, which has no bead-like structures, is connected with the cellular distribution of the pectic and extensin epitopes in the cortical and epidermal cells needs to be studied. If all these are proved, they would indicate an association between the scion, stock, and callus/graft union cells in sealing of the graft union. These aspects remain to be confirmed using other microscopy techniques including transmission electron microscopy.

## Conclusions

In the present study, analysis of the sectioned material indicated that grafting process was accompanied by deposition of extracellular material. SEM revealed two forms of the extracellularly deposited material – fibrillar and membranous – as well as the occurrence of bead-like structures on the surface of the graft union cells, long after a connection was established between the scion and stock. The extracellular material subsequently sealed the graft union. Only unesterified HG and extensins were detected abundantly in both the extracellular material and the bead-like structures. Thus, our results contribute to the current state of knowledge regarding plant grafting and introduce a structure, which is interesting in terms of its form and composition, that potentially protects the graft union area and which has not been observed during the grafting of other species before. The results also indicated a possibility of ionic interaction between the two polymers, acidic pectins and basic extensins, due to their chemical nature, which can result in the formation of a network with beneficial properties.

## Methods

### Plant material and sample preparation

Seeds of *Arabidopsis thaliana* Col-0 (Nottingham Arabidopsis Stock Center, Nottingham, UK; stock ID: N1093) were surface-sterilized with 20% commercial bleach (ACE Lever Co., Fater SpA, Pescara. Italy) for 8 min, rinsed five times with sterile distilled water (each time for 5 min), and left for 3 days at 4 °C in darkness. Then, the sterilized seeds were sown on Petri dishes with a medium containing ½ Murashige and Skoog salts (Sigma-Aldrich, St. Louis, MO, USA), 1% sucrose (CHEMPUR, Karlsruhe, Germany), and 0.8% agar (BioShop, Burlington, Ontario, Canada), pH 5.80–5.84. After seeding, the Petri dishes were sealed with parafilm (Bemis Company Inc., Neenah, WI, USA) and placed vertically in a growth chamber (20–22 °C, relative humidity 40%, photoperiod 16/8, and photosynthetic active radiation 40 μmol m^− 2^ s^− 1^). Following incubation, 4-day-old seedlings (six to seven seedlings per Petri dish) were transferred under sterile conditions onto Petri dishes with a medium containing ½ Murashige and Skoog salts (Sigma-Aldrich, St. Louis, MO, USA), 1% sucrose (CHEMPUR, Karlsruhe, Germany), and 1.8% agar (BioShop, Burlington, Ontario, Canada), pH 5.80–5.84. Later, another set of Petri dishes were prepared with a medium which was allowed to solidify at an angle of 15°, and grafting procedure was performed according to the method previously described [8] using an SZM-140 stereomicroscope (Motic, Hong Kong): First, horizontal cuts were made in the middle of the hypocotyls with a microknife (Fine Science Tools, Heidelberg, Germany), and then the scion and stock were carefully aligned together using a preparation needle. After grafting, the Petri dishes were sealed with parafilm (Bemis Company Inc., Neenah, WI, USA) and placed vertically in a growth chamber (20–22 °C, relative humidity 40%, photoperiod 16/8, and photosynthetic active radiation 40 μmol m^− 2^ s^− 1^).

The grafted seedlings were collected at 0–9 dag (60 seedlings on each day) and analyzed using an SZH10 stereomicroscope (Olympus, Tokyo, Japan). The roots, cotyledons, and developing leaves were excised, following which the grafted hypocotyls were fixed and embedded in Steedman’s wax as described previously [78]. Longitudinal sections (5- to 6-μm thick) were cut using a HYRAX M40 rotary microtome (Zeiss, Oberkochen, Germany) and collected on microscopic slides covered with Mayer’s albumin or coated with poly-L-lysine (Menzel Gläser, Braunscheig, Germany).

### Histo- and immunohistochemistry

The sections were dewaxed, rehydrated in a successive series of ethanol solutions (three times in 100% and once in 90 and 50% (*v*/v) solution, and then in distilled water; each wash for 10 min), and prepared for the histochemical analysis using a 0.05% (*w*/*v*) toluidine blue O aqueous solution used for general histological evaluation (Cat. No. T-0394; Sigma-Aldrich, St. Louis, MO, USA) and 0.5% (w/v) Sudan III solution used for detection of lipid substances (Cat. No. S4136; Sigma-Aldrich, St. Louis, MO, USA). Toluidine blue O is metachromatic dye that imparts different colors to particular cell components (e.g. green/blue for polyphenolic compounds, pink/purple for acidic polysaccharides with COO^−^ groups [79]), whereas Sudan III stains the lipids with an orange-red color [80].

For the immunolabeling procedure, the sections were dewaxed and rehydrated in a series of ethanol solutions (three times in 100, 90, and 50% (v/v) solution in phosphate-buffered saline (PBS); each wash for 10 min). The primary rat monoclonal antibodies (Plant Probes, Leeds, UK) used in the current study are listed in Table 2. The secondary antibody used was AlexaFluor 488 goat anti-rat antibody (Cat. No. 112–545-003; Jackson ImmunoResearch Laboratories, West Grove, PA, USA). The negative controls were prepared without the addition of primary antibody; hence, no fluorescence signal was observed in the control set of sections. Prior to immunolabeling with hemicellulose probes (LM15 and LM21 antibodies), the sections were incubated in pectate lyase (Cat. No. PRO-E0250; Prozomix Ltd., Northumberland, UK) and 3-(Cyclohexylamino)-1-propanesulfonic acid (CAPS) buffer (Cat. No. C263; Sigma-Aldrich, St. Louis, MO, USA) buffer solution to remove HG according to a procedure already described [81]. To visualize the cell walls, the sections were counterstained with 0.01% (*w*/*v*) Calcofluor White, a dye that stains cellulose (Fluorescent Brightener 28; Cat. No. F3543; Sigma-Aldrich, St. Louis, MO, USA), in PBS for 10 min. All the observations were performed and photographs were taken using a Nikon Eclipse Ni-U microscope equipped with a Nikon Digital DS-Fi1-U3 camera with the corresponding software (Nikon, Tokyo, Japan), at a maximum excitation wavelength of 490 nm (AlexaFluor 488) or 330 nm (Calcofluor White). The details of the staining procedures are described in previous studies [78, 82]. On each day of regeneration, five grafted hypocotyls were stained and their representative photographs were taken.

**Table 2:** List of primary rat monoclonal antibodies used in the current study

Antibody	Recognized epitope	References
*Hemicelluloses*		
LM15	XXXG motif of xyloglucan, shows some cross-reactivity with a single galactosyl residue in xyloglucan subunits XXLG and XLXG	[81]
LM21	β-(1 → 4)-manno-oligosaccharides in heteromannan (mannan, glucomannan, galactomannan polysaccharides)	[84]
*Pectins – homogalacturonan and rhamnogalacturonan I*		
LM19	unmethyl-esterified, partially methyl-esterified HG	[47]
LM20^a^	methyl-esterified HG	[47]
LM7^a^	partially methyl-esterified HG	[48]
LM8	xylogalacturonan, HG domain	[85]
LM5	linear tetrasaccharide in (1–4)-β-D-galactans (RG I side chain)	[86]
LM6	linear pentasaccharide in (1–5)-α-L-arabinans (RG I side chain)	[87]
LM13	longer stretches of 1,5-linked arabinosyl residues	[88]
LM16	epitope associated with arabinans, may involve galactosyl residue(s) on RG backbones	[89]
*AGPs*		
JIM8^b^	Arabinogalactan	[90]
JIM13	Arabinogalactan/ Arabinogalactan protein, carbohydrate epitope (β)GlcA1- > 3(α)GalA1- > 2Rha	[91]
LM2	Arabinogalactan protein, carbohydrate epitope containing β -linked GlcA	[92]
*Extensins*		
LM1^b^	Extensin/ HRGP (epitope most likely includes extensin glycan components)	[93]
JIM11^b^	Extensin/ HRGP glycoprotein	[94]
JIM12^b^	Extensin/ HRGP glycoprotein	[94]
JIM20^b^	Extensin/ HRGP glycoprotein	[94]

^a^Does not bind to unesterified HG. ^b^Epitope structure for carbohydrate antigen: unknown. GalA galacturonic acid, GlcA glucuronic acid, Rha rhamnose

### Scanning electron microscopy

For SEM analysis, the roots, cotyledons, and developing leaves were first excised from the grafted seedlings (4 and 9 dag). The remaining hypocotyls with the graft union zone were fixed by immediately placing them in 100% methanol (Sigma-Aldrich, St. Louis, MO, USA) for 30 min to 1 h, according to a previously described procedure [83]. After fixation, the samples were washed twice in 100% ethanol (each time for 30 min), followed by which ethanol was replaced with acetone. The dehydrated samples were dried with liquid carbon dioxide using a Pelco CPD 2 critical-point drier (Pelco, Fresno, CA, USA) and placed on aluminum stubs using double-sided adhesive carbon tape (Plano GmbH, Wetzlar, Germany). Then, the samples were coated with a 20-nm film of gold using a Pelco SC-6 sputter coater (Pelco, Fresno, CA, USA). The coated samples were observed using a Hitachi SU 8010 field emission SEM (Hitachi High-Technologies Corporation, Tokyo, Japan) with a secondary electron detector at accelerating voltages of 5 kV and 10 kV.

### Whole-mount and confocal microscopy

First, 4- and 9-dag hypocotyls were fixed in a mixture of 3% (*w*/*v*) paraformaldehyde (Polysciences, Washington, PA, USA) and 1.25% (*v*/v) glutaraldehyde (Sigma-Aldrich, St. Louis, MO, USA) in PBS, pH 7.2, overnight at 4 °C. Following fixation, the samples were washed three times with PBS (each time for 10 min) and placed in a blocking buffer containing 2% bovine serum albumin (Jackson ImmunoResearch Laboratories, West Grove, PA, USA) in PBS (w/v) for 30 min. After washing, the samples were incubated with primary antibodies (listed in Table 1) and with a secondary antibody (AlexaFluor 488) (Jackson ImmunoResearch Laboratories, West Grove, PA, USA). Following each incubation, the samples were washed three times with the blocking buffer (each time for 10 min). Then, the samples were rinsed three times with PBS (each time for 5 min), counterstained with 0.01% Calcofluor White (Fluorescent Brightener 28; Cat. No. F3543, Sigma-Aldrich, St. Louis, MO, USA) in PBS (w/v) solution, and rinsed with PBS and distilled water for three times (each time for 5 min). For each antibody, three hypocotyls were collected for analysis. The fluorescence of the Calcofluor White (excitation 365 nm, emission 435 nm) and secondary antibody (excitation 498 nm, emission 520 nm) was detected using an Olympus FV-1000 confocal system (Olympus, Hamburg, Germany) equipped with an Olympus IX81 inverted microscope, a 405-nm diode laser, and a multi-line argon ion laser (Melles Griot BV, Didam, Netherlands). A series of two-dimensional images of the optical sections through the hypocotyls (z-stacks) were taken using two separate photomultipliers. Image processing was performed using Fiji (ImageJ; NIH, Rockville, MD, USA).

### Photodocumentation

Photographs were obtained from two different channels (ultraviolet, blue light) using an epifluorescence and confocal microscope, and were combined using Fiji (ImageJ; NIH, Rockville, MD, USA). The figures (photographs and schemes) were assembled using CorelDrawX7 graphics program.

## Additional files

Additional file 1: **Figure S1.** Immunohistochemistry of grafted hypocotyl sections –homogalacturonan (LM7 and LM8 epitopes). **A** – lack of fluorescence signal. **A′** A, Calcofluor White. **B** – epitope detected in random sections (arrow) between groups of tracheary elements (asterisk) and other graft union cells. **C** – epitope present in some locations (arrow) within graft union area. **c** Calcofluor White. Scale bars: A and A′ = 50 μm; B and C = 10 μm (JPG 2277 kb)

Additional file 2: **Figure S2.** Immunohistochemistry of grafted hypocotyl sections – rhamnogalacturonan I (LM5, LM6, LM13, and LM16 epitopes). **A** and **B** – epitope detected abundantly in walls of graft union cells (arrows) and in low amount in walls of cortical cells (full arrow), epitope absent from extracellular material on the surface of graft union (arrowheads). **A′** A, Calcofluor White. **C and D** – epitope present in cellular compartments of graft union cells (arrows), no epitope observed in extracellular material on the surface of graft union (arrowheads). **C′** C, Calcofluor White. **E** – epitope detected in walls of some graft union cells (arrows), apart from extracellular material on the surface of graft union (arrowhead). **E′** E, Calcofluor White. **F** – strong fluorescence signal in cell wall of sieve tubes (arrows). **G** – epitope absent from graft union cells (arrows) and from extracellular material (arrowheads). **G′** G, Calcofluor White. **c** Calcofluor White. Scale bars: A, A′, C, C′, E, E′, G, and G′ = 50 μm; B, D, and F = 10 μm. (JPG 2868 kb)

Additional file 3: **Figure S3.** Immunohistochemistry of grafted hypocotyl sections – extensins (JIM12 and LM1 epitopes) and AGPs (JIM13, JIM8, and LM2 epitopes). **A** – epitope present in some of the cortical cells (full arrow) and graft union area (arrowheads), intensive fluorescence signal detected in the outer periclinal cell walls and cuticle of the epidermis (arrow); *inset:* intensive fluorescence signal detected in the outer periclinal cell walls and cuticle of the epidermis (arrow). **B** – epitope detected in the cell wall (arrow) and on the outside of the cell (arrowhead). **C** – epitope present in the cytoplasmic compartments of cortical cells near the graft union area (arrow). **D** – occurrence of epitope in the cells of the regenerated vascular bundle (arrows), in some endodermal cells (arrowhead), and peripheral cells of the graft union (*inset:* arrowhead), no fluorescence signal detected on the cell surface (full arrow). **E** – epitope present in the cytoplasm and/or plasmolemma of the graft union cells located peripherally (arrowheads), no fluorescence signal detected on the cell surface (arrow). **F** and *inset* – weak labeling in the cytoplasmic compartments of the peripheral cells (arrowheads), no fluorescence signal detected on the cell surface (arrows). **c** Calcofluor White, **ep** epidermis. Scale bars: A, D and *D inset*, and F = 50 μm; B, C, E, *A inset*, and *F inset* = 10 μm (JPG 2588 kb)

Additional file 4: **Figure S4.** Immunohistochemistry of grafted hypocotyl sections –xyloglucan (LM15 epitope), and heteromannan (LM21 epitope). **A** and **B** – abundant occurrence of the epitope in the walls of graft union cells (asterisks) except for endodermal or peripheral cells (arrows), no fluorescence signal detected on the graft union surface (full arrow). **A′** A, Calcofluor White. **C and D** – epitope detected in cellular compartments (arrows) and in the cell walls (full arrows) but not on the graft union surface (arrowheads). **C′** C, Calcofluor White. **c** Calcofluor White. Scale bars: A, A′, C, and C′ = 50 μm; B and D = 10 μm (JPG 3122 kb)

## Abbreviations

AGPs: arabinogalactan proteins
CAPS: 3-(cyclohexylamino)-1-propanesulfonic acid
dag: days after grafting
ECMSN: extracellular matrix surface network
HG: homogalacturonan
HRGPs: hydroxyproline-rich glycoproteins
PBS: phosphate-buffered saline
RG I: rhamnogalacturonan I
SEM: scanning electron microscope
