# Supplementary figures and images for: Unmethyl-esterified homogalacturonan and extensins seal Arabidopsis graft union
Source: BMC Plant Biol. 2019 Apr 18;19:151. doi: 10.1186/s12870-019-1748-4 (PMC6472031; doi:10.1186/s12870-019-1748-4)

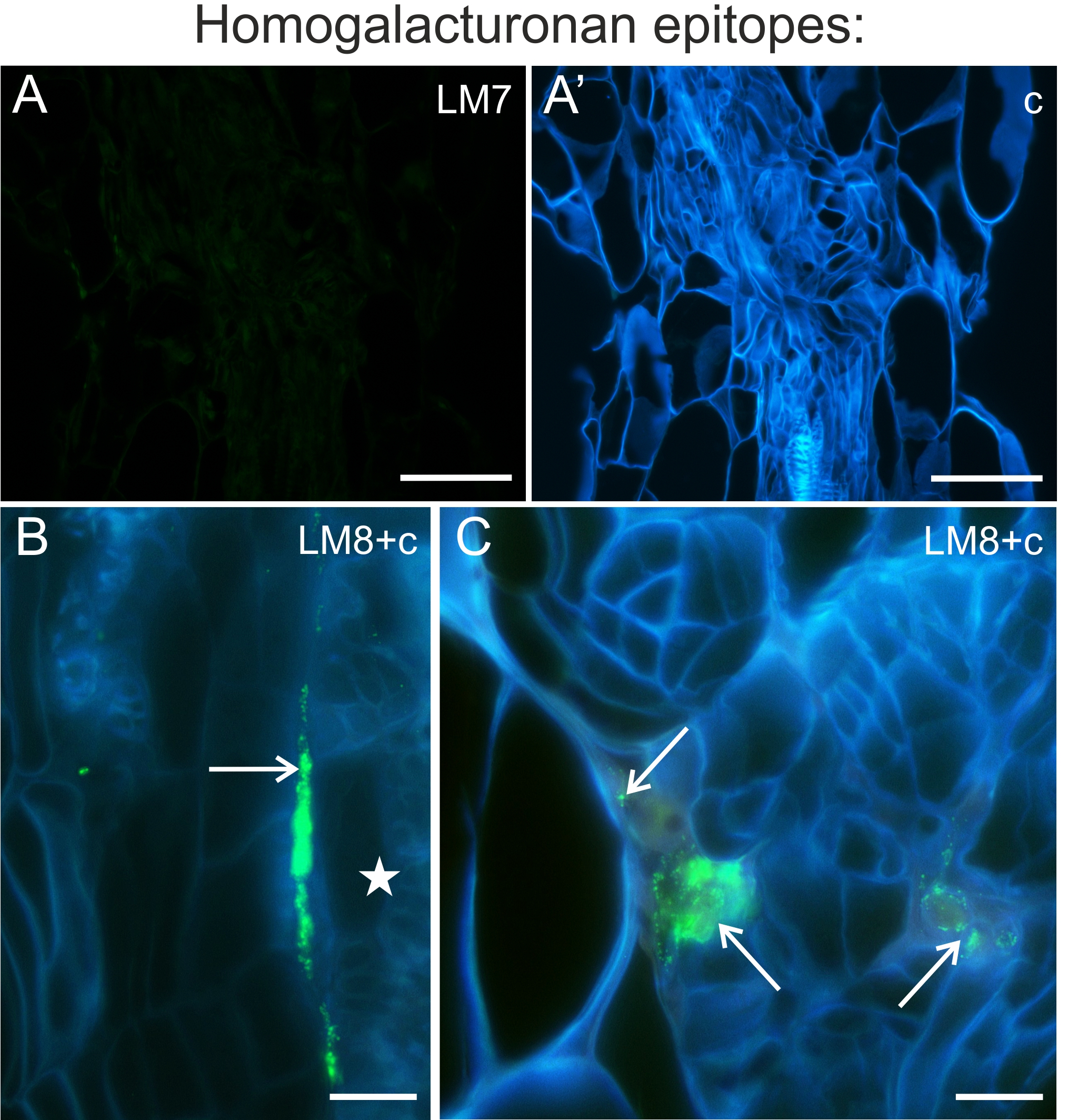

Supplement: Supplementary file 1 — Figure S1. Immunohistochemistry of grafted hypocotyl sections –homogalacturonan (LM7 and LM8 epitopes). A – lack of fluorescence signal. A′ A, Calcofluor White. B – epitope detected in random sections (arrow) between groups of tracheary elements (asterisk) and other graft union cells. C – epitope present in some locations (arrow) within graft union area. c Calcofluor White. Scale bars: A and A′ = 50 μm; B and C = 10 μm (JPG 2277 kb) [file 12870_2019_1748_MOESM1_ESM.jpg]

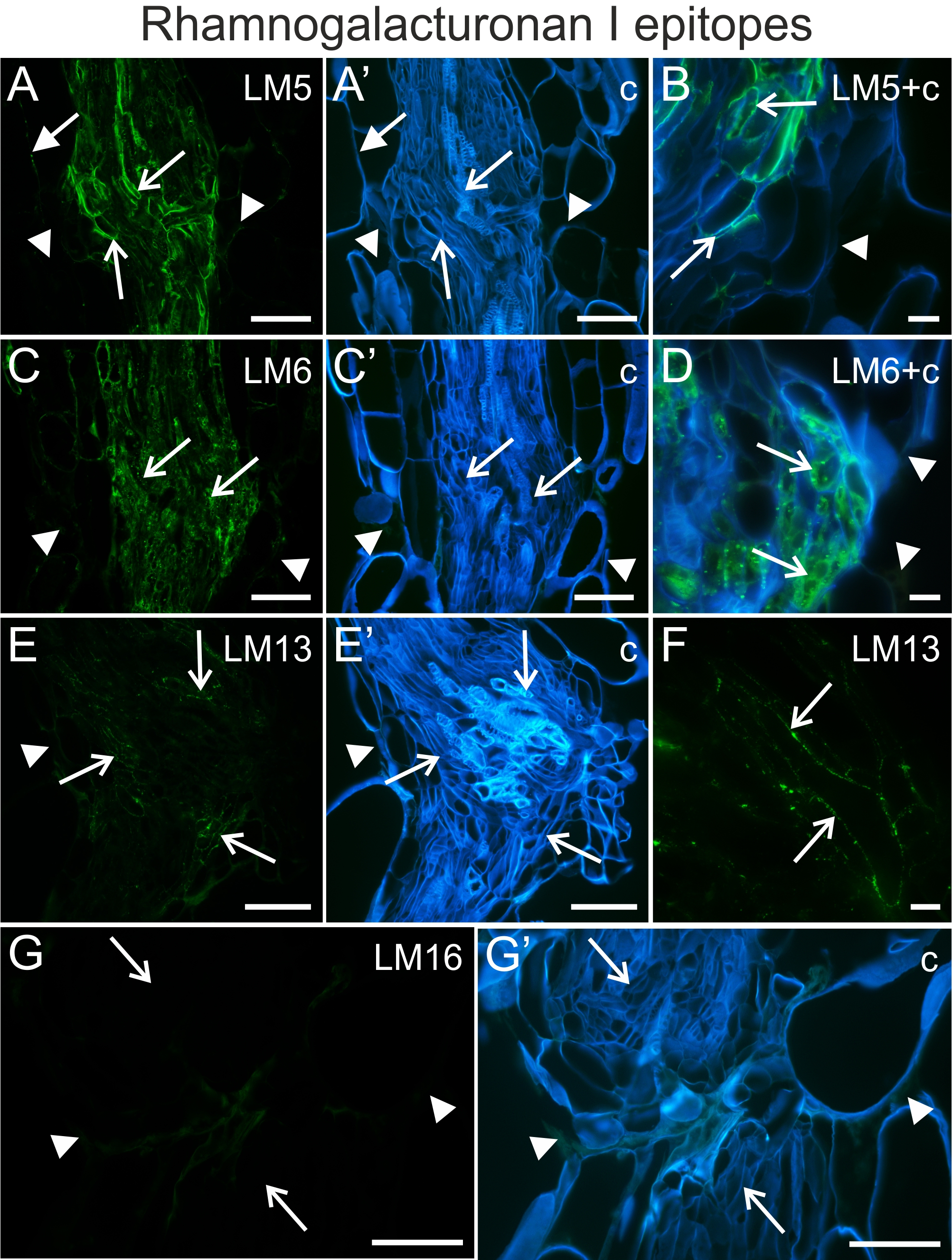

Supplement: Supplementary file 2 — Figure S2. Immunohistochemistry of grafted hypocotyl sections – rhamnogalacturonan I (LM5, LM6, LM13, and LM16 epitopes). A and B – epitope detected abundantly in walls of graft union cells (arrows) and in low amount in walls of cortical cells (full arrow), epitope absent from extracellular material on the surface of graft union (arrowheads). A′ A, Calcofluor White. C and D – epitope present in cellular compartments of graft union cells (arrows), no epitope observed in extracellular material on the surface of graft union (arrowheads). C′ C, Calcofluor White. E – epitope detected in walls of some graft union cells (arrows), apart from extracellular material on the surface of graft union (arrowhead). E′ E, Calcofluor White. F – strong fluorescence signal in cell wall of sieve tubes (arrows). G – epitope absent from graft union cells (arrows) and from extracellular material (arrowheads). G′ G, Calcofluor White. c Calcofluor White. Scale bars: A, A′, C, C′, E, E′, G, and G′ = 50 μm; B, D, and F = 10 μm. (JPG 2868 kb) [file 12870_2019_1748_MOESM2_ESM.jpg]

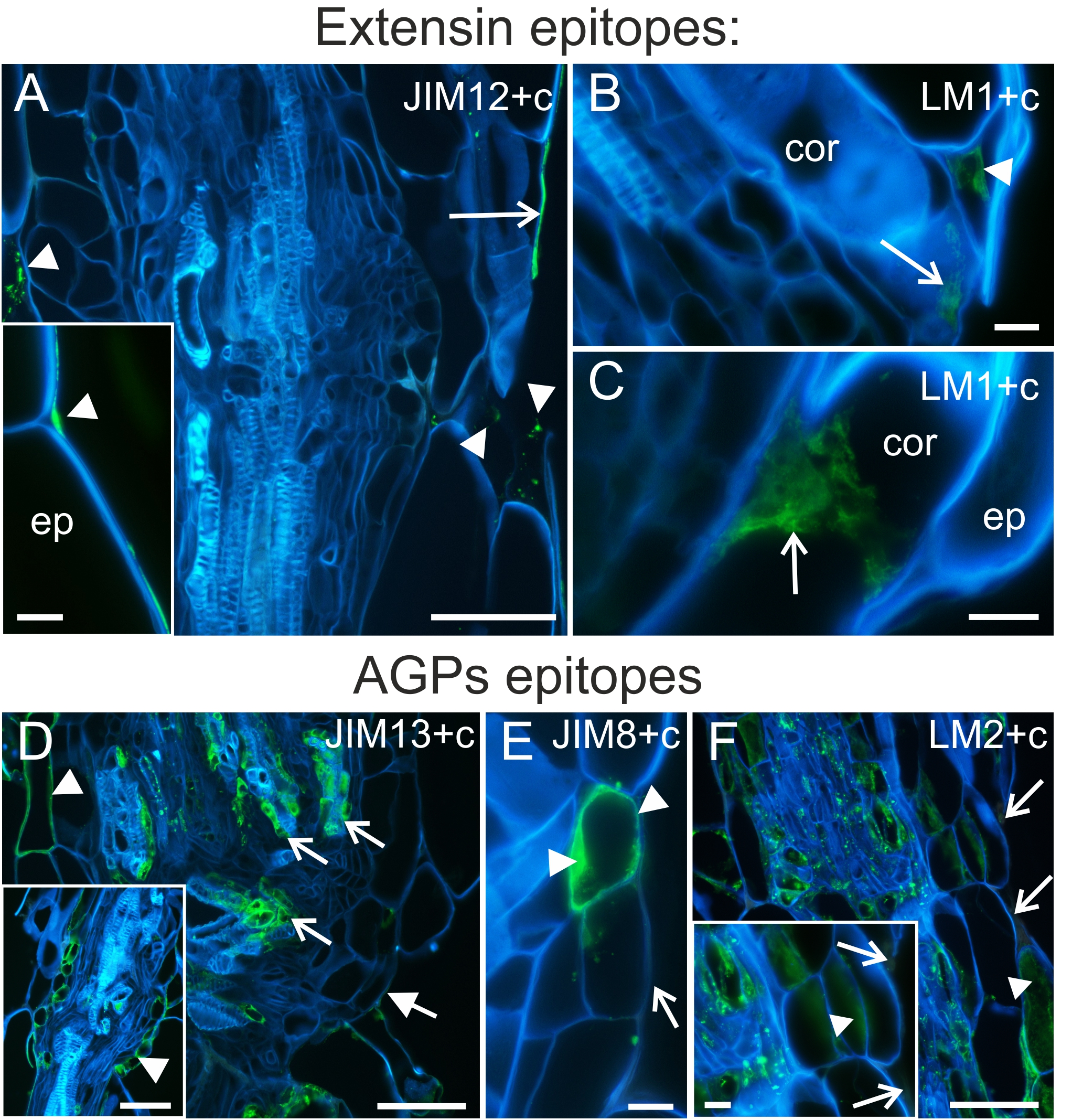

Supplement: Supplementary file 3 — Figure S3. Immunohistochemistry of grafted hypocotyl sections – extensins (JIM12 and LM1 epitopes) and AGPs (JIM13, JIM8, and LM2 epitopes). A – epitope present in some of the cortical cells (full arrow) and graft union area (arrowheads), intensive fluorescence signal detected in the outer periclinal cell walls and cuticle of the epidermis (arrow); inset: intensive fluorescence signal detected in the outer periclinal cell walls and cuticle of the epidermis (arrow). B – epitope detected in the cell wall (arrow) and on the outside of the cell (arrowhead). C – epitope present in the cytoplasmic compartments of cortical cells near the graft union area (arrow). D – occurrence of epitope in the cells of the regenerated vascular bundle (arrows), in some endodermal cells (arrowhead), and peripheral cells of the graft union (inset: arrowhead), no fluorescence signal detected on the cell surface (full arrow). E – epitope present in the cytoplasm and/or plasmolemma of the graft union cells located peripherally (arrowheads), no fluorescence signal detected on the cell surface (arrow). F and inset – weak labeling in the cytoplasmic compartments of the peripheral cells (arrowheads), no fluorescence signal detected on the cell surface (arrows). c Calcofluor White, ep epidermis. Scale bars: A, D and D inset, and F = 50 μm; B, C, E, A inset, and F inset = 10 μm (JPG 2588 kb) [file 12870_2019_1748_MOESM3_ESM.jpg]

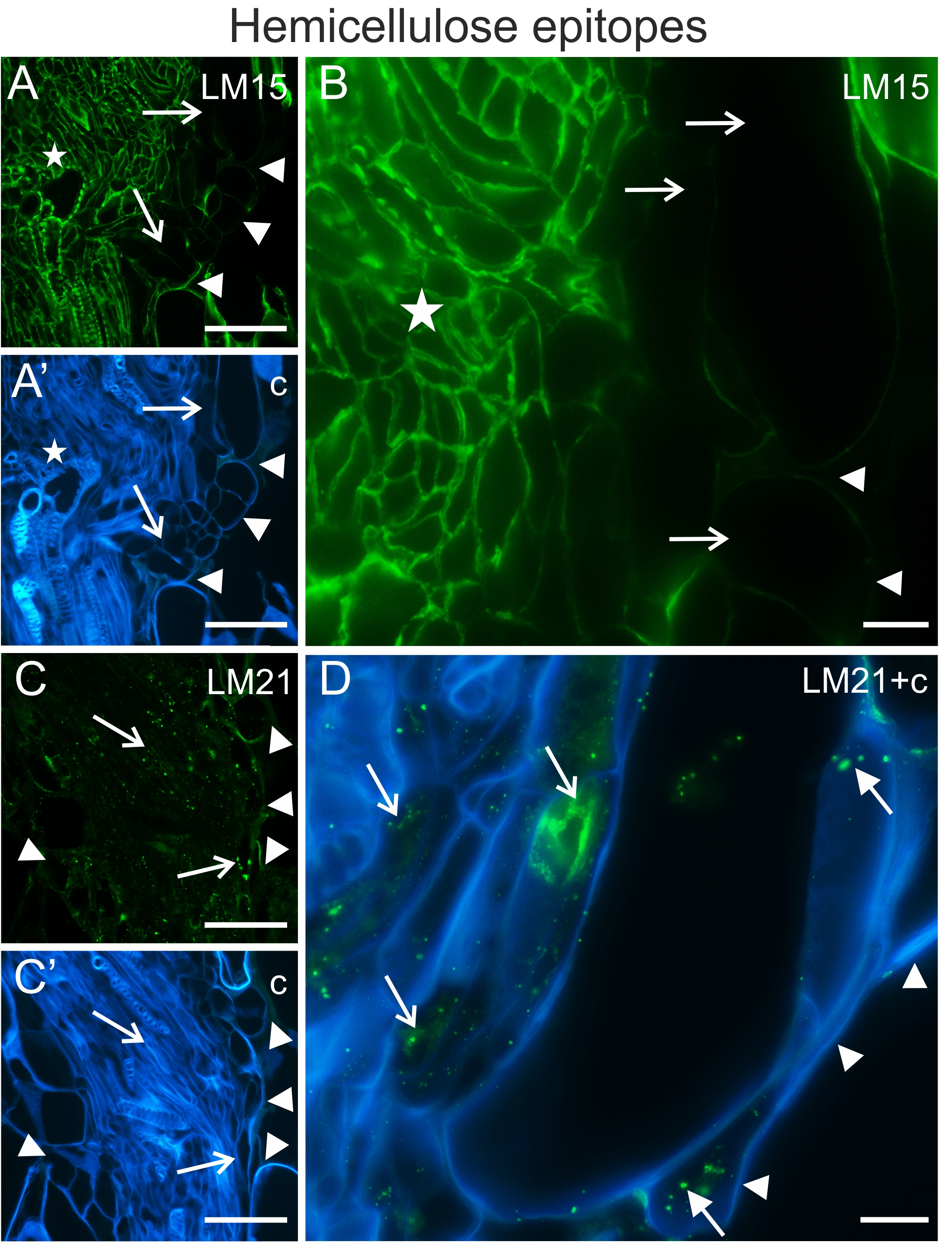

Supplement: Supplementary file 4 — Figure S4. Immunohistochemistry of grafted hypocotyl sections –xyloglucan (LM15 epitope), and heteromannan (LM21 epitope). A and B – abundant occurrence of the epitope in the walls of graft union cells (asterisks) except for endodermal or peripheral cells (arrows), no fluorescence signal detected on the graft union surface (full arrow). A′ A, Calcofluor White. C and D – epitope detected in cellular compartments (arrows) and in the cell walls (full arrows) but not on the graft union surface (arrowheads). C′ C, Calcofluor White. c Calcofluor White. Scale bars: A, A′, C, and C′ = 50 μm; B and D = 10 μm (JPG 3122 kb) [file 12870_2019_1748_MOESM4_ESM.jpg]
